# *Helicobacter pylori* and Inflammatory Bowel Disease: Unraveling the Complex Interactions and Clinical Implications

**DOI:** 10.1002/jgh3.70319

**Published:** 2025-12-28

**Authors:** Elaheh Karimzadeh‐Soureshjani, Farab Pourhasan, Pouria Ahmadi Simab, Nabi Jomehzadeh, Ali Saeedi‐Boroujeni

**Affiliations:** ^1^ Student Research Committee Abadan University of Medical Sciences Abadan Iran; ^2^ Clinical Research Development Unit Abadan University of Medical Sciences Abadan Iran; ^3^ Universal Scientific Education and Research Network (USERN) Tehran Iran; ^4^ Department of Microbiology, Faculty of Medicine Ahvaz Jundishapur University of Medical Sciences Ahvaz Iran; ^5^ Department of Basic Medical Sciences, Faculty of Medicine Abadan University of Medical Sciences Abadan Iran

**Keywords:** Crohn's disease, gut microbiota, *Helicobacter pylori*, inflammatory bowel disease, ulcerative colitis

## Abstract

*Helicobacter pylori* infection has been extensively studied in relation to various gastrointestinal disorders, with emerging evidence suggesting a significant association with inflammatory bowel disease (IBD). Epidemiological studies consistently demonstrate an inverse relationship between *H. pylori* infection and IBD development, particularly Crohn's disease (CD). Meta‐analyses reveal a significantly lower prevalence of *H. pylori* among IBD patients compared to healthy controls, supporting the hypothesis of a potential protective effect. This negative correlation appears particularly strong for virulent strains expressing *CagA*, suggesting strain‐specific immunomodulatory properties. The protective mechanisms may involve *H. pylori* 's ability to modulate host immune responses and maintain gut microbial homeostasis. Experimental models show that *H. pylori* colonization can induce regulatory T‐cell responses and downregulate pro‐inflammatory cytokines, potentially creating an immunological balance that protects against IBD development. Conversely, *H. pylori* eradication has been associated with increased IBD incidence and disease flares, possibly through disruption of established microbial ecosystems and immune regulation. Clinical observations further support this relationship, demonstrating that *H. pylori* ‐positive CD patients often experience milder disease courses with fewer complications. However, the interaction remains complex, as *H. pylori* infection may also exert detrimental effects in certain contexts. The bacterium's influence appears to depend on multiple factors, including infection timing, strain characteristics, and host genetic background. Current evidence highlights the crucial interplay between *H. pylori* , gut microbiota composition, and mucosal immunity in shaping IBD pathogenesis. Future research should focus on elucidating precise molecular mechanisms and evaluating whether targeted modulation of *H. pylori* could offer therapeutic potential, while considering potential risks.

## 1 Introduction

Inflammatory bowel disease (IBD), encompassing Crohn's disease (CD), ulcerative colitis (UC), and unclassified IBD (IBDU), is a group of chronic gastrointestinal disorders characterized by inflammation that can lead to serious complications like strictures and fistulae in CD [1]. Its global prevalence is steadily increasing, making it a significant healthcare burden [2]. Clinical presentations differ, with CD often involving diarrhea and abdominal cramping, while UC typically features bloody stools [3].

The pathogenesis of IBD is multifactorial, involving genetic susceptibility, environmental triggers, gut microbial dysbiosis, and immune dysfunction [4]. Notably, genetic factors account for only 19%–26% of heritability, underscoring the major influence of environmental and microbial determinants, even in low‐risk populations [5].

The intestinal microbiota, a vast and diverse ecosystem, is a critical determinant of health and disease [6, 7]. It contributes to physiology through nutritional processing, colonization resistance, and immunoregulation, including the establishment of immune tolerance [8]. In IBD, a characteristic dysbiosis occurs, marked by a depletion of Firmicutes and Bacteroidetes and an expansion of Proteobacteria [9]. This imbalance can enable typically harmless commensals, known as pathobionts, to become pathogenic, influencing IBD's initiation and progression [5]. These insights have spurred investigation into the potential crosstalk between *Helicobacter pylori* ( *H. pylori* ) colonization and gut microbial ecology in IBD pathogenesis [10].

*H. pylori* is a gram‐negative, spiral‐shaped bacterium that colonizes the gastric mucosa [11]. With a long co‐evolutionary history with humans, its global prevalence is high, especially in developing nations [12, 13]. It is the primary etiological agent of gastroduodenal pathologies, including chronic gastritis, peptic ulcer disease, and gastric cancer [14]. The bacterium's pathogenicity is driven by virulence factors like *CagA* and *V* *acA*, which sustain gastric inflammation [15]. Notably, *H. pylori* 's clinical significance extends beyond the stomach. It has been detected in extra‐gastric sites and implicated in various systemic manifestations, including hematological and autoimmune conditions [16, 17, 18]. A key characteristic of this pathogen is its ability to establish long‐term infection and chronic inflammation through immunomodulatory capabilities [19].

This has stimulated research into its potential relationship with Inflammatory Bowel Disease (IBD). The bacterium's dual role in immune regulation and its capacity to modify gut microbial ecology are of particular interest for understanding IBD development mechanisms [20]. Therefore, investigating the *H. pylori* ‐IBD relationship offers significant pathogenic insights and could pave the way for novel, microbiome‐based therapeutic approaches for IBD management.

## 2 Gastrointestinal Microbiome and *Helicobacter pylori*

*H. pylori* colonization significantly influences gut microbial ecology [21]. Its infection is strongly correlated with gastric microbiome perturbations that can promote dysbiosis and subsequent gastric pathology [22]. While the dominant bacterial phyla are consistent in *H. pylori* ‐positive and negative individuals, their relative abundances vary significantly. These *H. pylori* ‐induced alterations are partially reversible, as antimicrobial eradication therapy often restores gastric microbial richness. However, the pathogen's effects on distal colonic microbiota remain poorly characterized [23].

As part of this complex ecosystem, *H. pylori* interacts with gut microbiota through direct bacterial crosstalk and host‐mediated mechanisms [24]. Its strategies to alter microbiota composition include secreting pathogenic effectors, stimulating antimicrobial peptide production, competitive exclusion for resources, and modulating pH and the immune response [25]. Although its gastric niche is well‐defined, emerging evidence suggests it influences microbial populations throughout the gastrointestinal tract, potentially establishing new ecological equilibria in the large intestine [20].

Clinical studies confirm these systemic effects. Benavides‐Ward et al. reported elevated *Proteobacteria*, *Firmicutes*, and *Prevotella* in *H. pylori* ‐positive subjects [26], while Lino et al. found altered *Lactobacillus* profiles [27]. Gao et al. identified significant fecal dysregulation, particularly of *Bacteroidetes*, *Firmicutes*, and *Proteobacteria* [28]. Notably, the *Nitrospirae* phylum is exclusively found in *H. pylori* ‐negative subjects, likely due to nitrite's antimicrobial activity against the pathogen. These compositional shifts occur even in asymptomatic carriers, with specific microbial increases observed in both pediatric and adult populations [29].

Eradication therapy for *H. pylori* induces transient perturbations in gut microbial ecology. Studies on bismuth‐based quadruple therapy report a temporary decline in microbial diversity, though this may be partly attributable to concomitant proton pump inhibitors and bismuth salts [30]. One investigation noted decreases in *Firmicutes*, *Bacteroidetes*, *Verrucomicrobia*, and *Lentisphaerae*, with increases in *Proteobacteria* and *Cyanobacteria*. While these shifts largely resolved by day 56 post‐treatment, certain taxonomic alterations persisted, including elevated Enterobacteriaceae and *Leuconostocaceae* and reduced *Rikenellaceae*, *Christensenellaceae*, *Peptococcaceae*, *Clostridiales* Family *XI*, and *Victivallaceae* [31]. These findings highlight that the observed microbial changes are therapy‐associated rather than solely due to *H. pylori* eradication. This underscores the need to understand the pathogen's dual impact on gastric and colonic communities to elucidate gastrointestinal disease mechanisms. Consequently, there is potential clinical value in developing adjunct strategies to maintain or restore gut microbial homeostasis during anti‐ *H. pylori* treatment protocols.

## 3 Inflammatory Bowel Diseases (IBD) and Gastrointestinal Microbiome

The precise etiology of Inflammatory Bowel Disease (IBD) remains incompletely understood, arising from a complex interplay of genetic susceptibility, immunological dysfunction, and modifiable lifestyle factors [32]. A central paradigm in its pathogenesis is intestinal dysbiosis a significant perturbation in the gut microbiota's structure and function [33]. The prevailing model posits that IBD manifests from pathological interactions between the gut microbiota and host immune defenses, exacerbated by impaired intestinal barrier integrity and dysregulated immune responses [32].

Specific microbial populations are strongly implicated; pro‐inflammatory species like *Enterococcus faecalis* and *Clostridium septicum* are linked to disease chronicity, whereas short‐chain fatty acid (SCFA)‐producing bacteria confer anti‐inflammatory, mucosal protection [34]. Compared to healthy controls, IBD patients consistently exhibit reduced microbial diversity and density, characterized by a depletion of Firmicutes and Bacteroidetes phyla and a marked expansion of Proteobacteria and Actinobacteria [20]. A clinically significant feature is the pronounced loss of specific commensal species vital for maintaining inflammatory homeostasis.

Microbiota‐derived metabolites, specifically short‐chain fatty acids (SCFAs) and bile acids, are fundamental to inflammatory bowel disease (IBD) pathogenesis. SCFAs, produced from microbial fermentation of dietary fibers, exert multifaceted effects including enhancing mucosal regulatory T cells, inhibiting neoplastic proliferation, protecting against experimental colitis, modulating macrophage function, and regulating energy homeostasis via gut hormone secretion [35].

Modulation of bile acid homeostasis can expand colonic regulatory T cells and ameliorate colitis. Ursodeoxycholic acid (UDCA) shows promise in improving barrier integrity and attenuating inflammation in preclinical models, though its therapeutic efficacy in IBD requires further clinical validation [36]. Among SCFAs, sodium butyrate demonstrates dual antimicrobial and immunomodulatory properties, inhibiting *H. pylori* proliferation and virulence while suppressing NF‐κB‐mediated inflammation. Conversely, the metabolite TMAO promotes *H. pylori* survival and pathogenicity [37]. Clinically, SCFA levels transiently decrease during eradication therapy but recover significantly post‐treatment [38]. These findings underscore the complex interplay between microbial metabolites and host immunity, highlighting promising avenues for microbiome‐targeted interventions in IBD management.

## 4 *Helicobacter pylori* and Immune System

To establish gastric colonization, *H. pylori* must overcome gastric acidity and host immunity. It achieves this through flagella‐mediated motility to penetrate the mucosal layer and urease activity, which neutralizes acid and modifies mucus viscosity. Its pathogenicity is mediated by virulence factors such as *CagA*, *VacA*, outer membrane vesicles, *OipA*, *HtrA*, and *NapA* [39]. Among these, *CagA* and *VacA* are particularly noteworthy; *CagA* is linked to severe clinical outcomes and alters host cell signaling, while *VacA* induces pleiotropic effects including apoptosis and immunomodulation. Both factors cause cytoskeletal rearrangements and can trigger autoantibody production [40].

Following epithelial colonization, *H. pylori* initiates a defined immune cascade: (1) proinflammatory cytokine release from epithelial cells, (2) immune cell infiltration into the mucosa, and (3) subsequent cytokine amplification. This immunostimulatory environment facilitates antigen presentation, eliciting both cellular and humoral immune responses that can be analyzed through the frameworks of pathogen recognition, innate immune activation, and adaptive immunity development.

The host immune response to *H. pylori* begins with Pattern Recognition Receptors (PRRs) on epithelial and immune cells detecting bacterial Pathogen‐Associated Molecular Patterns (PAMPs) [41]. A key virulence mechanism is the Type IV Secretion System (T4SS), which injects the *CagA* effector and bacterial LPS precursor into host cells, activating signaling cascades like TRAF and immune responses [42]. *H. pylori* LPS can also act as a superantigen.

**FIGURE 1:**
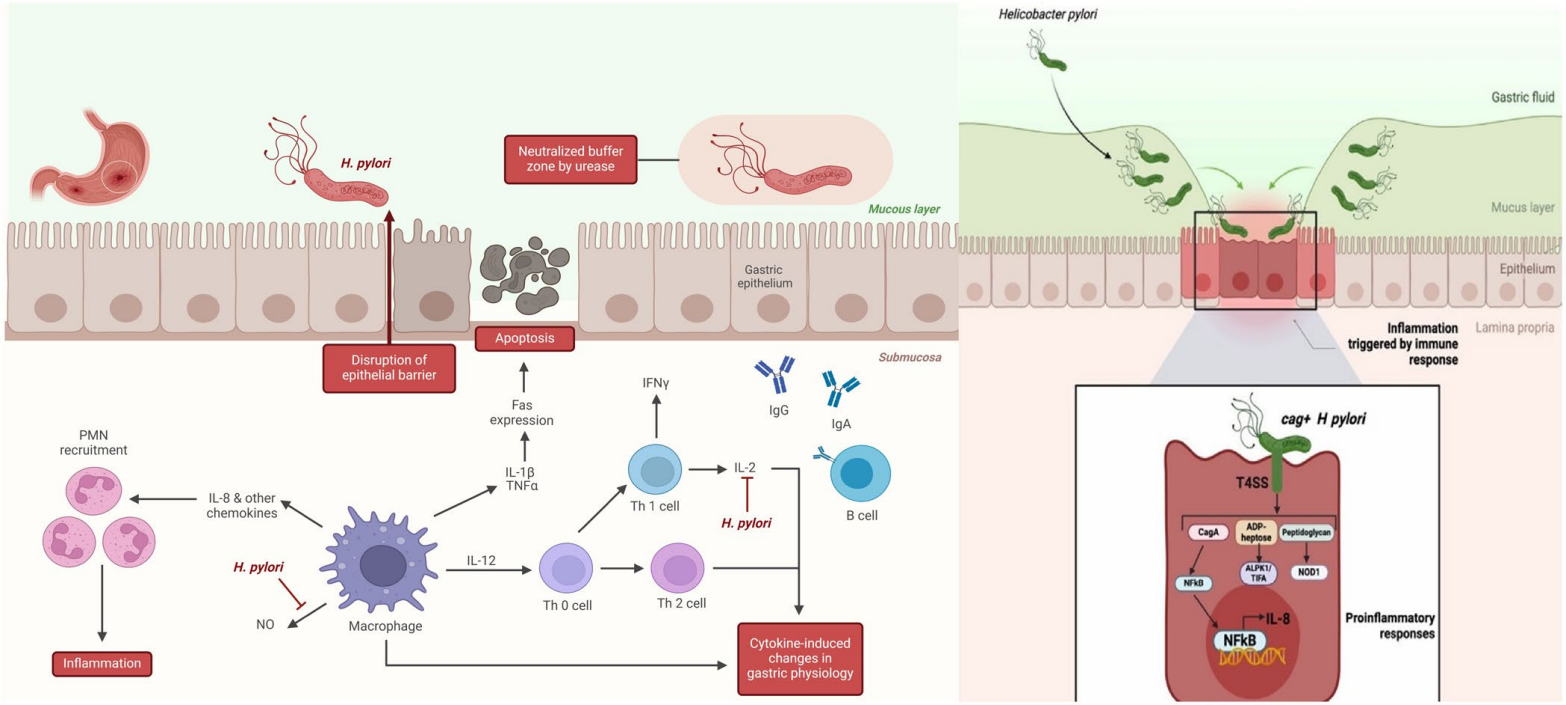
The inflammatory response induced by *Helicobacter pylori* (*H. pylori*) begins with the bacterium's colonization of the gastric epithelium. By producing urease, *H. pylori* neutralizes the acidic gastric environment, facilitating its adhesion to the mucosal surface. After attachment, it injects the *CagA* protein into host cells, triggering the production of pro‐inflammatory cytokines, including IL‐8, IL‐1, and TNF‐α. These cytokines recruit neutrophils (PMNs) and macrophages to the infection site. Additionally, the activation of T‐helper cells and subsequent release of IFN‐γ further amplify and sustain the inflammatory cascade. This figure was created by the authors using BioRender.

Innate immunity is triggered as infected epithelial cells release chemokines (e.g., IL‐8, CXCL, CCL20), recruiting neutrophils, monocytes, and macrophages to the infection site [43]. For the adaptive phase, dendritic cells in Peyer's patches present antigens, priming naïve T cells [44]. Activated CD4+ T cells localize to the lamina propria and differentiate into Th1 or Th2 subsets, with *H. pylori* ‐reactive CD4+ T cells consistently found in the gastric mucosa of infected individuals [45] (Figure 1).

*H. pylori* infection triggers distinct T‐cell responses and cytokine profiles. Bacterial components like urease stimulate B‐cells to produce autoantibodies (e.g., anti‐dsDNA, IgM rheumatoid factor) [46]. Immune activation follows bacterial adhesion, leading to cytokine secretion and specific humoral immunity. Molecular mimicry is a key mechanism; for instance, the homology between the urease β subunit and host parietal cell ATPase can induce cross‐reactive autoimmune responses [47]. Infected individuals exhibit elevated proinflammatory cytokines (IFN‐γ, TNF‐α, IL‐1) [48], and IFN‐γ may promote APC recognition of parietal cells via cross‐reactive epitopes, potentially causing apoptosis [49].

**FIGURE 2:**
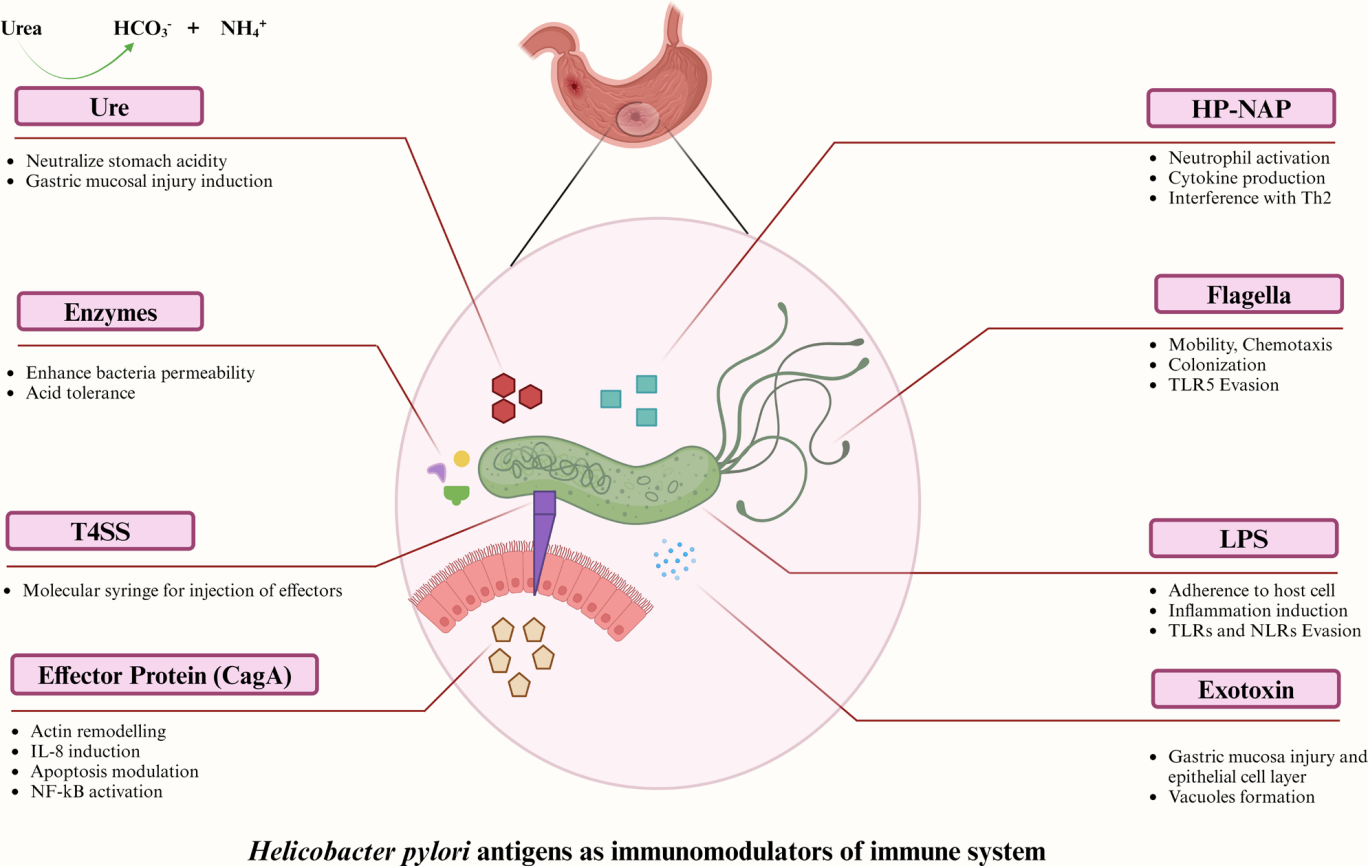
*H. pylori* employs multiple strategies to establish infection and drive disease pathogenesis. To survive the stomach's acidic environment, it utilizes urease, arginase, and heat shock proteins (HSPs). The bacterium then uses flagella‐mediated motility and various adhesins to reach, adhere to, and colonize the gastric epithelium. Once attached, it damages host cells through effector proteins and toxins such as *VacA*. Furthermore, *H. pylori* actively potentiates the inflammatory response via specific virulence factors. BabA, blood group antigen‐binding adhesion; *CagA*, cytotoxin‐associated gene A; HP‐NAP, neutrophil‐activating protein; IL‐8, interleukin‐8; LPS, lipopolysaccharide; NF‐κβ, nuclear factor kappa‐light‐chain‐enhancer of activated B cells; OipA, outer inflammatory protein A; ROS, reactive oxygen species; SabA, sialic acid‐binding adhesion; SOD, superoxide dismutase; T4SS, type IV secretion system; TLR5, Toll‐like receptor 5; Ure, urease; VacA, vacuolating cytotoxin A. This figure was designed by the authors using BioRender.

To facilitate chronicity, *H. pylori* employs immune evasion strategies, including structural modification of surface antigens (LPS, flagellin) to reduce immunogenicity [50] and virulence factor‐mediated modulation of T‐cell responses [51]. Understanding these immunomodulatory interactions is crucial, particularly regarding IBD predisposition, and may reveal novel therapeutic targets for immune dysregulation in *H. pylori* ‐positive IBD patients. A summary of these immune interactions is provided in Table 1, and major virulence factors are depicted in Figure 2.

**TABLE 1:** Immune response mechanisms and evasion strategies of *Helicobacter pylori* .

Category	Description	Key components
*H. pylori* survival mechanisms	Mechanisms *H. pylori* uses to survive in the acidic environment of the stomach.	Flagella: Promotes movement in the stomach lining. Urease: Converts urea to CO_2_ and ammonia; alters mucus viscosity. Toxins/Proteins: Includes *CagA*, *VacA*, *OMV*, *OipA*, *HtrA*, *OMP*, *NepA*.
Immune system activation	How *H. pylori* activates the immune response in the host.	Pattern Recognition Receptors (PRRs): On gastric epithelial cells. Type IV Secretion System (T4SS): Transports *CagA* and other factors into host cells. Antigen Recognition: Initiates immune response.
Innate immune response	The initial immune response to *H. pylori* infection.	PRRs recognize pathogen‐associated molecular patterns (PAMPs). Neutrophils, monocytes, macrophages recruited to infection site. Chemokines: IL‐8, CXC chemokine ligand, CC chemokine ligand 20.
Adaptive immune response	The specific immune response involving B cells and T cells.	Antigen‐specific B and T cells appear. T Cells: Th1 and Th2 responses. B Cells: Secrete anti‐DNA antibodies, IgM rheumatoid factor. Molecular Mimicry: Similarities between *H. pylori* antigens and gastric tissues.
Immune evasion strategies	Strategies employed by *H. pylori* to evade the host immune response.	Antigen Modification: Alteration of surface antigens like LPS and flagella. Regulation of T Cell Responses: Through virulence factors.

*Note:* *H. pylori Survival Mechanisms:* This section describes the methods *H. pylori* uses to survive in the acidic environment of the stomach. *Immune System Activation:* This section explains how *H. pylori* activates the host's immune response. *Innate Immune Response:* This part covers the initial immune response to *H. pylori* infection. *Adaptive Immune Response:* Details the specific immune response involving B cells and T cells. *Immune Evasion Strategies:* Discusses the strategies *H. pylori* employs to evade the host immune response.

## 5 Inflammatory Bowel Disease and *H. pylori*

### 5.1 *H. pylori* 's Role in Modulating Inflammatory Bowel Disease

An inverse epidemiological relationship exists between *H. pylori* colonization and Inflammatory Bowel Disease (IBD), suggesting the bacterium's survival strategies may confer protective immunomodulatory effects. This complex duality is explained by several immunological frameworks. The distinct immune polarizations in Crohn's disease (Th1) and ulcerative colitis (Th2 or mixed) may modify the host response to *H. pylori* , and conversely, chronic *H. pylori* infection may systemically reprogram immune reactivity [52].

Molecular analyses show significant upregulation of multiple cytokines (e.g., IFN‐γ, TNF, IL‐1β, IL‐8, IL‐10) in *H. pylori* ‐infected gastric epithelium [53]. Following TLR‐mediated recognition, dendritic cells can polarize T‐cell responses toward either a pro‐inflammatory Th1 phenotype (via IL‐12) or an immunoregulatory Th2/Treg phenotype (via IL‐10), illustrating the immunological plasticity that underlies this dual role [54].

**FIGURE 3:**
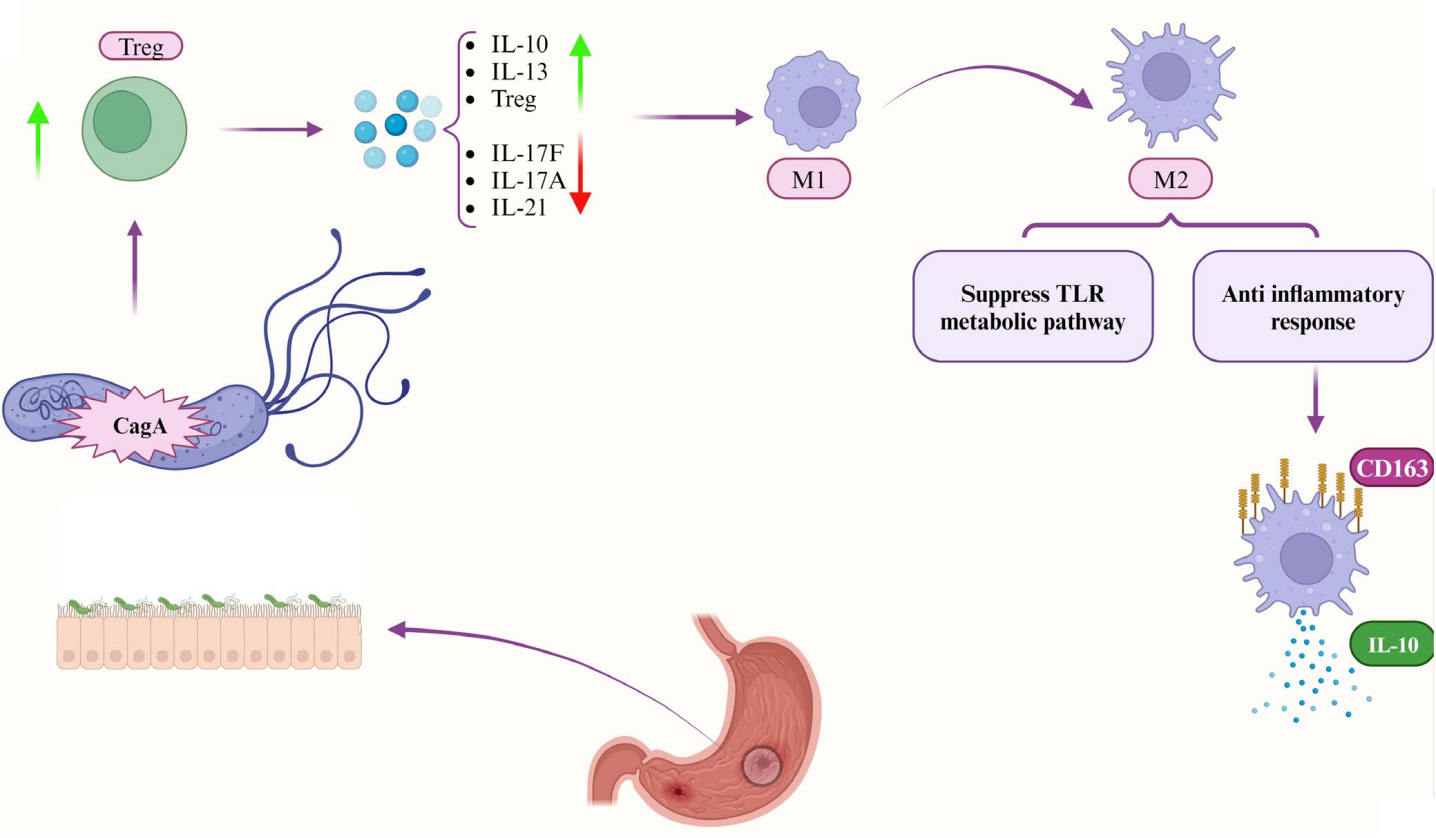
The presence of the cytotoxin‐associated antigen A (*CagA*) gene in *Helicobacter pylori*'s Cag pathogenicity island is associated with a more favorable prognosis in inflammatory bowel diseases (IBD) through a series of immunomodulatory mechanisms. First, *CagA* induces a shift in the host immune response by modulating the balance between T helper 17 (Th17) cells and regulatory T cells (Tregs). This modulation reduces the levels of pro‐inflammatory cytokines IL‐17F, IL‐17A, and IL‐21 while increasing the expression of anti‐inflammatory cytokines IL‐13 and IL‐10, along with Treg populations. Together, these cytokine changes act synergistically to promote the conversion of pro‐inflammatory M1 macrophages to the anti‐inflammatory M2 lineage. The resulting M2 macrophages then suppress signaling mediated by Toll‐like receptors (TLRs) and activate specific metabolic pathways via the transcription factor BATF2, which increases expression of the scavenger receptor CD163. This process further amplifies the production of IL‐10. The overall increase in IL‐10 and the sustained anti‐inflammatory environment ultimately lead to a protective effect, resulting in a better clinical prognosis for IBD. BATF2, Basic leucine zipper transcription factor ATF‐like 2; *CagA*, cytotoxin‐associated antigen A; *H. pylori*, *Helicobacter pylori*; IL, Interleukin; TLR, Toll‐like receptor; Treg, Regulatory T cell; Th17, T helper 17. This figure was designed by the authors using BioRender.

Substantial evidence supports a protective mechanism whereby *H. pylori* infection promotes regulatory T cell (Treg) activation while inhibiting Th1 and Th17 responses [55]. Tregs are pivotal for maintaining intestinal immune equilibrium, demonstrated by their capacity to prevent or treat experimental colitis in adoptive transfer models [56] and by the spontaneous colitis that develops in IL‐10‐deficient mice lacking functional Treg populations [57]. The mechanisms of *H. pylori* 's *CagA* antigen in IBD prognosis are depicted in Figure 3.

Further clinical and experimental evidence elucidates the mechanisms behind *H. pylori's* potential protective role in IBD. Clinically, *H. pylori* ‐positive IBD patients show reduced systemic type I interferon levels [58]. Experimentally, oral delivery of *H. pylori* DNA ameliorates colitis in murine models by suppressing dendritic cell production of pro‐inflammatory mediators like type I interferon and IL‐12 [58]. Similarly, *H. pylori* SS1‐colonized mice exhibit attenuated colitis, associated with elevated IL‐10 and suppressed Th17 responses [59]. Additional protective mechanisms may include cross‐reactive antibody production that offers protection against related pathogens [60] and the reduction of gastric acidity, creating an unfavorable environment for other potential pathogens.

This inverse *H. pylori* ‐IBD relationship is also framed by the hygiene hypothesis, where improved sanitation reduces *H. pylori* transmission, potentially impairing proper immune education and increasing IBD risk [61, 62, 63]. Furthermore, genetic factors modulate this interaction; a population‐based study found that carriers of the Crohn's disease risk allele (A) in the *ATG16L1* T300A variant (*rs2241880*) displayed altered susceptibility to *H. pylori* infection, suggesting autophagy‐related genetic variation influences these host‐microbe interactions [64]. This indicates that early‐life *H. pylori* exposure might mitigate IBD risk in genetically predisposed individuals through immune programming.

### 5.2 Epidemiological Relationship Between *H. pylori* and IBD

A 2018 comprehensive review established a significant inverse relationship between *H. pylori* infection and the incidence of all IBD subtypes (CD, UC, IBDU) across diverse geographic regions [65]. Supporting this, Tanner et al. found that patients with gastritis, duodenitis, or PUD had a lower prevalence of *H. pylori* if they also had IBD [66]. Furthermore, *H. pylori* eradication therapy was associated with a decreased likelihood of developing IBD within 5 years post‐treatment. These findings hold significant clinical relevance for the ongoing debate on universal eradication therapy, including for asymptomatic infections [67]. Epidemiological studies predominantly indicate an inverse association between *H. pylori* infection and Inflammatory Bowel Disease (IBD) incidence [68, 69, 70]. This protective correlation is particularly noted for Crohn's Disease (CD) [71]. Meta‐analyses consistently report a lower *H. pylori* prevalence in IBD and CD patients compared to healthy controls [72, 73, 74], a finding corroborated by broader reviews [75, 76]. A large‐scale meta‐analysis confirmed this significant inverse association but highlighted substantial heterogeneity, influenced by demographic and environmental factors such as age and geographic region [77]. Therefore, the evidence suggests a potentially protective, rather than definitively causal, relationship. Future multicenter studies controlling for confounders are necessary to elucidate this association further.

### 5.3 *H. pylori* and IBD‐Related Markers

Research indicates that *H. pylori* infection may confer a protective effect against Inflammatory Bowel Disease (IBD) by modulating immune and inflammatory pathways. Understanding the specific molecular mechanisms and biomarkers involved is crucial to elucidating this potential role. Key immune components, including the *CagA* virulence factor, interleukins (e.g., IL‐18, IL‐10), and immune cell regulation, are central to determining whether *H. pylori* exerts a protective or pathogenic influence in IBD [78].

#### 5.3.1 Role of *CagA* in Relation to IBD

The *cytotoxin‐associated gene A* (*CagA*) is a major *H. pylori* virulence factor translocated into host cells via a type IV secretion system (T4SS). While implicated in gastric carcinogenesis, *CagA* also possesses immunomodulatory properties relevant to immune‐mediated disorders like IBD [79].

*CagA*‐positive *H. pylori* strains are associated with a reduced risk of IBD, particularly Crohn's disease (CD). This protective effect is attributed to *CagA*'s ability to modulate host immunity, such as suppressing pro‐inflammatory cytokines like TNF‐α [79]. A meta‐analysis of 1748 subjects confirmed a significant inverse relationship, showing *CagA* seropositivity reduced overall IBD risk by 69% and CD risk by 75% [79].

The observed effects are associated with *CagA*‐positive strains in general. However, the specific molecular variations, such as EPIYA motif types and the integrity of the *cag pathogenicity island* (*cagPAI*) which critically influence host signaling were not detailed in the cited studies, warranting cautious interpretation [79].

#### 5.3.2 Immune Responses and Inflammatory Markers

Following *H. pylori* infection, the host's cytokine response is pivotal to its potential protective role in inflammatory bowel disease (IBD). The pro‐inflammatory cytokine IL‐18 contributes to immune regulation by enhancing regulatory T cell (Treg) activity, thereby helping to prevent chronic intestinal inflammation. Concurrently, *H. pylori* stimulates the production of the anti‐inflammatory cytokine IL‐10, which shifts immune polarization away from pathogenic Th1/Th17 pathways; however, the causal involvement of IL‐10 requires further experimental confirmation [54]. Conversely, the infection can attenuate key pro‐inflammatory mediators like TNF‐α, IFN‐γ, and IL‐12, thereby modulating the broader inflammatory network and potentially mitigating the escalation of intestinal inflammation characteristic of IBD.

#### 5.3.3 Impact on Other Inflammatory Markers

Beyond *CagA*, *H. pylori* infection modulates key inflammatory markers relevant to Inflammatory Bowel Disease (IBD), particularly by influencing dysregulated Th1 and Th17 immune pathways. It downregulates these pro‐inflammatory responses, characterized by cytokines like IFN‐γ and TNF‐α, thereby attenuating the chronic mucosal inflammation central to Crohn's disease and ulcerative colitis. In parallel, the infection promotes an immunological shift that favors the expansion and function of regulatory T cells (Tregs). This enhancement of Treg activity helps restore immune homeostasis by countering pro‐inflammatory mechanisms, which is instrumental in mitigating excessive immune activation and subsequent tissue damage in the gastrointestinal tract.

#### 5.3.4 Genetic Factors and Influence on Response to *H. pylori*

Genetic predisposition, particularly through polymorphisms in autophagy‐related genes like *ATG16L1*, is a critical determinant of susceptibility to both *H. pylori* infection and Inflammatory Bowel Disease (IBD) [64]. Risk alleles in *ATG16L1*, which impair autophagic function, can compromise immune defenses against pathogens like *H. pylori* and contribute to dysregulated intestinal inflammation, thereby increasing IBD risk [64]. Conversely, early‐life exposure to *H. pylori* may counteract this genetic susceptibility and confer a protective effect against IBD. The dynamic interplay between host genetic architecture and environmental exposures such as *H. pylori* colonization helps explain the heterogeneity in IBD development, clarifying why some at‐risk individuals remain protected while others progress to disease.

### 5.4 *H. pylori* Eradication and Inflammatory Bowel Disease (IBD): Guidelines and Research Findings

Based on the 2017 ACG guidelines, testing for and treating *H. pylori* is recommended for patients with conditions like peptic ulcer disease and a history of gastric cancer, with meta‐analyses suggesting eradication may reduce gastric cancer risk [80, 81]. This practice coincides with an epidemiological transition marked by a rising incidence of Inflammatory Bowel Disease (IBD) in Western populations alongside a declining prevalence of *H. pylori* [82]. Data from the Taiwan National Health Insurance Research Database indicates that *H. pylori* eradication therapy may be associated with an elevated risk of developing autoimmune disorders, including IBD, raising concerns about its broader immunological consequences [83]. However, the relationship remains complex; while some evidence suggests eradication could contribute to IBD relapse, several studies, including one by Shinzaki et al. and a case–control study aligned with Rosania's findings, reported no significant short‐term change or effect on IBD disease activity following eradication [84, 85]. Furthermore, research by Zhong et al. revealed no definitive association between the infection and IBD subtypes, indicating a potentially indirect role in pathogenesis [76].

A growing body of literature explores the complex association between *H. pylori* eradication and the onset or relapse of inflammatory bowel disease (IBD). While eradication therapy reduces all‐cause mortality in peptic ulcer disease patients, it has been concurrently linked to an increased incidence of autoimmune conditions, including IBD, particularly in younger individuals [86]. Clinical case reports further support this, documenting the onset of ulcerative colitis (UC) shortly after eradication in both adult and pediatric patients [87, 88, 89]. These instances suggest that *H. pylori* may contribute to immune homeostasis in certain hosts, and its removal can disrupt this balance, potentially triggering IBD. Conversely, in established Crohn's disease, *H. pylori* infection has been associated with a milder clinical phenotype, characterized by fewer complications like fistulas and strictures, indicating a potential modulatory influence on disease progression [90].

Consequently, the impact of *H. pylori* eradication on Inflammatory Bowel Disease (IBD) is multifaceted and context‐dependent. While evidence suggests a protective effect of the bacterium against disease severity and complications, other studies associate its eradication with an increased risk of disease relapse or exacerbation. This divergence may be influenced by the timing of eradication, as intervention during early life when the gut microbiome is more plastic could cause more profound and lasting ecological shifts than in adulthood. These contrasting findings underscore the necessity for a patient‐specific approach in clinical management. A nuanced risk‐benefit analysis is therefore essential, weighing the goals of *H. pylori* eradication against the potential implications for immunological and gastrointestinal health, particularly in individuals with or predisposed to IBD. A summary of this empirical data is provided in Table 2.

**TABLE 2:** Empirical evidence on the relationship between *H. pylori* eradication and inflammatory bowel disease (IBD).

Evidence	Summary	Conclusion	Key point	References
ACG Clinical Guidelines (2017)	Testing for *H. pylori* is recommended for patients with peptic ulcer disease, low‐grade MALT lymphoma, and a history of endoscopic resection of early gastric cancer. Treatment is essential if the test result is positive.	Diagnosing and treating *H. pylori* is important to prevent complications and gastric cancer.	Need for treatment in patients with a history of gastric issues.	[80]
Lee et al. (2016). Meta‐analysis (24 studies)	Treating asymptomatic *H. pylori* infection in adults may reduce the risk of gastric cancer.	Treating *H. pylori* can effectively reduce the risk of gastric cancer.	Emphasis on the importance of treating *H. pylori* to lower cancer risk.	[81]
National Health Insurance Database in Taiwan	Treatment for *H. pylori* infection is associated with an increased risk of autoimmune diseases, including IBD.	Caution is needed in managing *H. pylori* treatment, especially in patients at risk for IBD.	Increased risk of autoimmune diseases as a result of *H. pylori* treatment.	[83]
Zhong et al. (2021)	*H. pylori* infection does not correlate with the type or classification of IBD, and eradication of *H. pylori* may lead to IBD relapse.	Eradicating *H. pylori* may have negative effects on IBD relapse.	No direct correlation between *H. pylori* and type of IBD.	[76]
Shinzaki et al. (2018)	Eradicating *H. pylori* does not affect the short‐term disease activity of IBD.	Eradicating *H. pylori* may have limited effects on short‐term disease activity.	Limited impact on disease activity in the short term.	[84]
Rosania et al. (2018). Case–control study (127 patients)	This study compared 127 IBD patients (90 with Crohn's disease and 37 with ulcerative colitis) and showed similar results regarding the impact of *H. pylori* .	The effects of *H. pylori* treatment in IBD patients are confirmed.	Detailed comparisons between patients and controls.	[85]
Sheu et al. (2022)	*H. pylori* treatment increases the incidence of autoimmune diseases and IBD while decreasing all‐cause mortality in patients with peptic ulcer disease.	*H. pylori* treatment may lead to an increased incidence of IBD, especially in younger patients.	Treatment effects are more significant in younger patients.	[86]
Chiba et al. (2016)	A report of ulcerative colitis occurring immediately after *H. pylori* eradication, indicating a direct relationship between treatment and disease onset.	*H. pylori* treatment can suddenly lead to the onset of ulcerative colitis.	Immediate effects of *H. pylori* treatment on IBD occurrence.	[87]
Homolak et al. (2021)	A report of a 72‐year‐old woman who developed ulcerative colitis after *H. pylori* eradication, highlighting clinical and endoscopic changes.	There is a relationship between *H. pylori* treatment and the onset of colitis in older individuals.	Treatment of *H. pylori* impacts disease onset in older adults.	[88]
Fujita et al. (2021)	A report of a 12‐year‐old boy with ulcerative colitis who relapsed after *H. pylori* treatment, despite improvements in duodenal ulcer symptoms and endoscopic findings.	*H. pylori* treatment may lead to IBD relapse in some patients.	Possibility of IBD relapse after *H. pylori* treatment.	[89]
Fialho et al. (2019)	*H. pylori* infection is independently associated with less fistulizing/stricturing disease and less active colitis in Crohn's disease patients.	*H. pylori* infection may help reduce complications in Crohn's disease patients.	Positive association between *H. pylori* infection and reduced complications in CD patients.	[90]

### 5.5 How *H. pylori* May Influence the Clinical Course of IBDs

The precise mechanisms by which *H. pylori* exerts a protective effect against inflammatory bowel diseases (IBDs) remain to be fully elucidated. Nevertheless, a growing number of investigations, particularly those employing murine models, have provided valuable insights into the underlying immunological processes. Zhang et al. [91] demonstrated that colonization by *H. pylori* could confer protection against chronic dextran sulfate sodium (DSS)‐induced colitis by modulating the Th17/Treg immune balance and inducing macrophage polarization toward the anti‐inflammatory M2 phenotype. Complementing this, Chen et al. [92] showed that serum‐derived exosomes from *H. pylori* ‐infected patients with chronic gastritis promoted the upregulation of NLRP12 in intestinal epithelial cells. This upregulation inhibited the Notch signaling pathway, resulting in the decreased expression of intestinal chemokines such as MCP‐1 and MIP‐1α, thereby implicating immune regulatory pathways in the bacterium's protective role against IBD. Further supporting evidence was provided by Gravina et al. [93], who reported that Hp [2, 3, 4, 5, 6, 7, 8, 9, 10, 11, 12, 13, 14, 15, 16, 17, 18, 19, 94], a formyl peptide receptor (FPR) agonist derived from *H. pylori* , accelerated mucosal healing in a rat model of TNBS‐induced colitis. This therapeutic effect correlated with a significant reduction in colonic levels of pro‐inflammatory mediators, including COX‐2, TGF‐β, TNF‐α, and tissue transglutaminase (t‐TG). Similarly, Kao et al. [95] found that *H. pylori* infection shifts the dendritic cell (DC)‐mediated polarization of Th17/Treg responses toward a Treg‐dominant phenotype, which may attenuate Th17‐mediated immune activation and thereby offer protection against the development of IBD.

The protective effects attributed to *H. pylori* , particularly strains expressing the *CagA* virulence factor, are thought to stem from the bacterium's immunomodulatory capabilities. These effects facilitate both antigen‐specific and nonspecific immune responses within the intestinal environment, contributing to the mitigation of dysbiosis and inflammation. This immunomodulation is particularly relevant for individuals with a genetic predisposition to autoimmune diseases, not only restricted to the gastrointestinal tract but also encompassing conditions such as asthma, allergic rhinitis, and other allergic disorders [96]. Several hypotheses propose that the pathogenesis of IBD may be linked to an imbalance between pathogenic and commensal bacteria, alongside a disruption in the equilibrium of pro‐ and anti‐inflammatory immune responses. Furthermore, insufficient exposure to enteric pathogens during early childhood, which typically induce episodes of gastroenteritis, has been implicated as a potential risk factor for developing IBD [65].

Experimental models of colitis in mice have demonstrated that a single symbiotic microorganism is capable of triggering colitis, which may parallel mechanisms relevant to human IBD [97]. Moreover, chronic infection with *H. pylori* has been shown to induce significant alterations in the composition of the large intestinal microbiota, suggesting that *H. pylori* may exert regulatory effects on the gut microbial community, thereby influencing IBD onset and progression. Clinical interventions such as probiotic administration and fecal microbiota transplantation have further supported this concept by demonstrating notable improvements in IBD treatment outcomes [98]. As a result, the recurrence rate of IBD tends to be elevated following *H. pylori* eradication, likely due to disruption of the finely tuned gut microbial balance. Martin‐Nunez et al. [99] reported that both *H. pylori* infection and its antibiotic‐mediated eradication induce shifts in gut microbiota composition that may predispose patients to IBD relapse.

Eradication of *H. pylori* disrupts the intestinal microbiota equilibrium, inducing dysbiosis akin to that observed in inflammatory bowel disease (IBD). This disruption is characterized by diminished microbial diversity and a decline in populations of short‐chain fatty acid (SCFA)‐producing bacteria, notably members of the *Ruminococcaceae* family, while potentially fostering the proliferation of pathogenic species. Such microbial shifts may result in reduced availability of essential energy substrates for epithelial cells, impaired differentiation of regulatory T cells (Tregs), degradation of the intestinal mucus barrier, mucosal inflammation, and alterations in mucosal permeability. Consequently, the recurrence of IBD following *H. pylori* eradication may be attributable, at least in part, to these microbiota disturbances.

Although the exact protective mechanisms of *H. pylori* against IBD remain to be fully elucidated, evidence from murine models highlights regulated Th17/Treg cell responses, modulation of NLRP12 expression, and decreased levels of pro‐inflammatory mediators such as COX‐2, TGF‐β, TNF‐α, and tissue transglutaminase (t‐TG) as plausible explanations. Importantly, the role of the intestinal microbiota in IBD pathogenesis is unequivocal. Given the substantial impact of *H. pylori* on gut microbial composition and the notable changes observed after its eradication, the gut microbiome is likely a critical mediator in the interplay between *H. pylori* infection and IBD development.

*H. pylori* infection represents a significant risk factor for gastric cancer and is implicated in a spectrum of upper gastrointestinal disorders, including peptic ulcer disease, atrophic gastritis, gastric adenomas, and gastric mucosa‐associated lymphoid tissue (MALT) lymphoma. Furthermore, epidemiological evidence has linked *H. pylori* infection to various extra‐gastric conditions, such as ischemic heart disease, neurodegenerative disorders, and hematologic abnormalities including iron deficiency anemia, immune thrombocytopenic purpura, and vitamin B12 deficiency [100]. These data strongly reinforce the necessity of *H. pylori* eradication, even in asymptomatic individuals. Although the associations between inflammatory bowel disease (IBD) and *H. pylori* infection are compelling, current evidence is insufficient to justify withholding eradication therapy. Consequently, the advantages of *H. pylori* eradication surpass the potential risks related to IBD, and clinical management should continue to align with established protocols for the treatment of *H. pylori* infection.

## 6 Conclusion and Future Prospective

*H. pylori* infection, a key gastric pathogen, exhibits a complex and paradoxical relationship with inflammatory bowel diseases (IBD). While its colonization is historically associated with a decreased IBD risk, recent evidence indicates that both its presence and eradication can significantly influence IBD onset, progression, and recurrence.

The bacterium may confer protection by modulating host immunity, potentially through the induction of tolerogenic dendritic cells and regulatory T cells (Tregs) that suppress pro‐inflammatory pathways. Conversely, *H. pylori* eradication, particularly in genetically susceptible individuals, has been linked to an increased risk of IBD, likely due to antibiotic‐induced dysbiosis. This creates a clinical dilemma, as the recommended universal eradication strategy, endorsed for gastric cancer prevention, may inadvertently disrupt immune homeostasis and elevate autoimmune risks.

Therefore, future studies must prioritize elucidating the specific immunological and microbial mechanisms involved. A shift toward personalized medicine is essential, focusing on identifying genetic, microbial, and environmental factors that determine individual outcomes. This will enable the development of tailored strategies that maximize the benefits of *H. pylori* eradication while mitigating potential adverse effects on IBD risk, ultimately leading to more precise and effective clinical management.

## Funding

The authors declare that no funds, grants, or other support were received during the preparation of this manuscript.

## Ethics Statement

This study was reviewed and approved by Abadan University of Medical Sciences with the approval number: IR.ABADANUMS.REC.1402.123. Ethical issues, such as data fabrication, double publication and submission, redundancy, plagiarism, consent to publish, and misconduct, have been checked by all the authors before publication in this journal.

## Conflicts of Interest

The authors declare no conflicts of interest.

## Data Availability

The data that support the findings of this study are available from the corresponding author upon reasonable request.
